# Modelling of combination therapy using implantable anticancer drug delivery with thermal ablation in solid tumor

**DOI:** 10.1038/s41598-020-76123-0

**Published:** 2020-11-09

**Authors:** Muneer Al-Zu’bi, Ananda Mohan

**Affiliations:** Centre for Health Technologies, Faculty of Engineering and Information Technology, University of Technology Sydney, Sydney, NSW 2007 Australia

**Keywords:** Computer modelling, Biotechnology, Cancer, Computational biology and bioinformatics, Systems biology, Molecular medicine, Oncology

## Abstract

Local implantable drug delivery system (IDDS) can be used as an effective adjunctive therapy for solid tumor following thermal ablation for destroying the residual cancer cells and preventing the tumor recurrence. In this paper, we develop comprehensive mathematical pharmacokinetic/pharmacodynamic (PK/PD) models for combination therapy using implantable drug delivery system following thermal ablation inside solid tumors with the help of molecular communication paradigm. In this model, doxorubicin (DOX)-loaded implant (act as a transmitter) is assumed to be inserted inside solid tumor (acts as a channel) after thermal ablation. Using this model, we can predict the extracellular and intracellular concentration of both free and bound drugs. Also, Impact of the anticancer drug on both cancer and normal cells is evaluated using a pharmacodynamic (PD) model that depends on both the spatiotemporal intracellular concentration as well as characteristics of anticancer drug and cells. Accuracy and validity of the proposed drug transport model is verified with published experimental data in the literature. The results show that this combination therapy results in high therapeutic efficacy with negligible toxicity effect on the normal tissue. The proposed model can help in optimize development of this combination treatment for solid tumors, particularly, the design parameters of the implant.

## Introduction

Cancer is one of the most dangerous and deadliest diseases that cause deaths of millions of people around the world each year. More than 85% of human cancers appear as solid tumors^1^. Minimally invasive and image-guided thermal ablation techniques such as radiofrequency ablation (RFA) and high-intensity focused ultrasound (HIFU) ablation have been used for local treatment of malignant solid tumors as an alternative for systemic chemotherapy and surgical resection^2^. In thermal ablation, the tissue temperature rises above 50 °C, which is enough to destroy tumor tissue by inducing coagulation necrosis^2^. Thus, thermal ablation will destroy the cellular and vasculature structures, which results in a new ablated tissue with different characteristics. However, the main limitation and challenge of thermal ablation is the risk of local recurrence and residual tumor after the thermal ablation, particularly at the tumor periphery^3–5^. The experimental studies showed that the combination therapy through local release of anticancer drugs from a miniaturized implant in solid tumors following thermal ablation can result in a better therapeutic efficacy by destroying the residual cancer cells and preventing tumor recurrence^6–8^.

Anticancer drug distribution and fluid flow within solid tumors are essential factors that affect the clinical efficiency of anticancer therapies. In addition to that, pharmacokinetic processes, including drug efflux/influx into cells, drug binding/unbinding with interstitial proteins, perfusion into blood capillaries, and biodegradation, have a significant impact on the therapeutic outcomes. Moreover, one of the most important challenges in the development of new drug delivery systems (DDSs) is ensuring that the optimal amount of drug is achieved in tumor versus normal tissues to avoid toxicity in the healthy tissues. Dual-release implants have been clinically approved and currently used for cancer treatments in the clinical trials and research, e.g., Gliadel polymer implants^9^. The implant releases the anticancer drug over two phases, namely, burst and sustained releases. For example, in-situ forming implants (ISFIs) have a dual-release pattern with a large undesirable burst release, which may cause major toxicity problems and consume the loaded drug in the implants rapidly^10^. Designing this type of implants with a minimum initial burst release becomes an attractive challenge, and one of the key issues in the design of ISFIs^11^. Moreover, some drug-loaded implants can be designed in a controlled way to have a dual-release profile to release a large amount of drug early, i.e., burst release, to rapidly reach the effective therapeutic concentration at the target site while keeping drug concentration within the effective level during the sustained release phase^12^. Therefore, providing mathematical pharmacodynamic (PD) and pharmacokinetic (PK) models to examine and analyze the impact of the anticancer drug on the surrounding healthy and tumor tissues is necessary for design the implantable DDSs to get high therapeutic efficiency.

Mathematical and computational modelling of release and transport of anticancer drugs have played a vital role in the advancement of drug delivery systems. These models provide a powerful tool for understanding drug transport and other complex pharmacokinetics processes and their impact on the tumor cells and the surrounding tissues (pharmacodynamics). As a result, they can help in optimum design and development of the DDSs to reduce the number of animal experiments which save time and reduce cost. A comprehensive review of literature has been discussed on mathematical models that have been employed to improve and design anti-cancer DDSs^13–15^. For example, mathematical models were used to aid the design and optimization of doxorubicin-loaded liposome formulations to achieve a better therapeutic index in tumor^16^. Application of mathematical modeling to guide the development of various drug delivery systems, e.g., extended-release formulation, liposome, etc., was presented^17^. These models optimized the drug formulation and dose regimen, accelerated the clinical trial, evaluated the influence of the drug on anti-tumor efficacy, predicted the clinical response by preclinical data, etc. A generic model is developed to minimize the number of suppositions about drug distribution to describe the behavior of therapeutic and diagnostic drugs in tumor environments^18^. Furthermore, a mathematical model is developed to study the effect of the various factors on the delivery of BCNU chemotherapy to brain tumor using systemic administration and local release from Gliadel wafer^19^. This model yields information on the optimal polymer implant location and the efficacy of controlled drug delivery by Gliadel wafer compared to traditional degradable polymers.

Most of the mathematical works on anticancer drug transport are limited to systemic drug delivery while few simplified models on implantable drug delivery systems (IDDSs) following thermal ablation are reported^12,20,21^. A mathematical model was derived at steady-state for design dual-release doxorubicin (DOX)-loaded implant to provide the optimal drug pharmacokinetics at the tumor ablation boundary after RFA^12^. A numerical model was proposed to estimate the DOX drug transport parameters, e.g., diffusivity and elimination rate, following insertion of a dual-release implant in liver tissues with/without RFA^22^. A computational transport model was proposed for simulation and prediction transport and pharmacokinetics of DOX after inserting biodegradable implants in liver tumors following RFA^20,21,23^. However, the models discussed above are derived based on many simplified assumptions, such as taking the impact of pharmacokinetics (e.g., elimination, cellular uptake/efflux, and binding) as an average equilibrium process via an effective rate constant. These models do not characterize and predict the dynamic intracellular concentration of anticancer drugs and the binding of anticancer drugs with proteins in the tissue. Furthermore, the works mentioned above do not provide any pharmacodynamic model which can be used for evaluating the therapeutic efficacy, i.e., the impact of the anticancer drug on tumor and healthy tissues.

**Figure 1:**
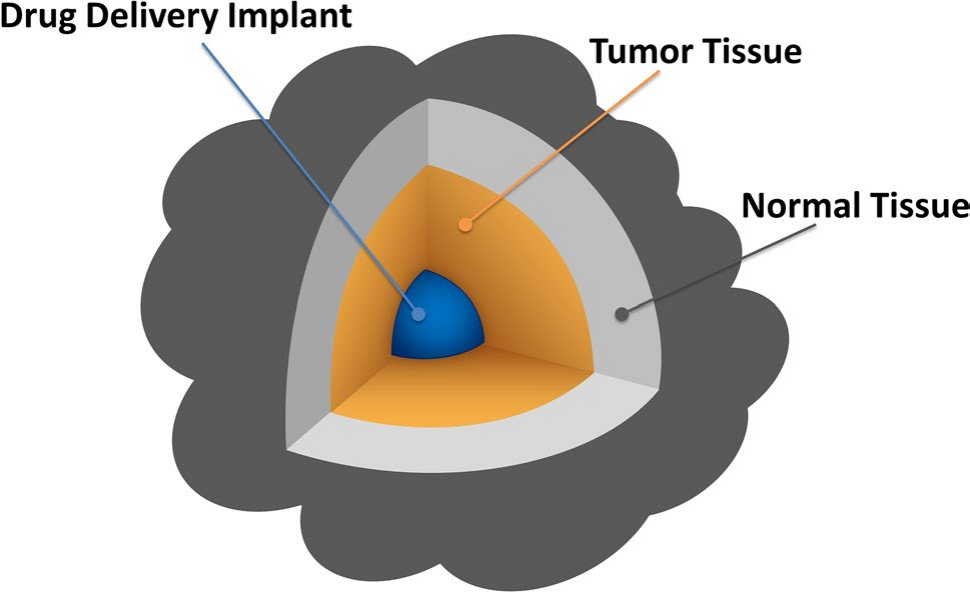
Graphical illustration of the drug implant inserted in 3-D solid tumor.

**Figure 2:**
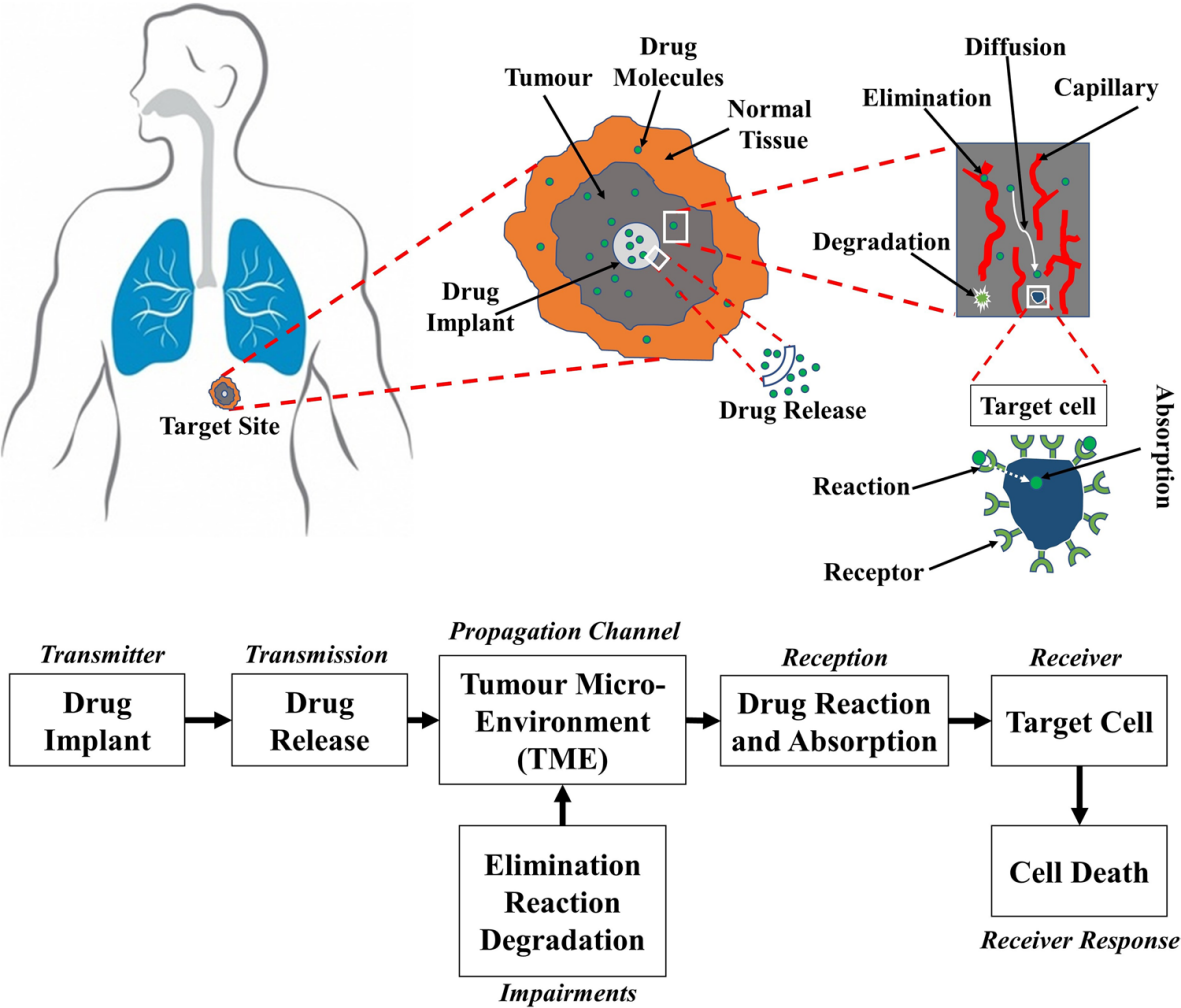
Abstraction of the implantable drug delivery system in tumor using molecular communication paradigm.

In this paper, we develop a comprehensive mathematical and computational model for local release and transport of anticancer DOX drug following insertion of a dual-release implant inside a thermally ablated solid tumor with the help of molecular communication (MC) abstraction, see Fig. 1. We chose DOX anticancer drug because it is widely used in chemotherapy due to its efficacy in killing a wide range of cancers such as carcinomas, sarcomas, and hematological cancers^24^. Moreover, there are many experimental measured parameters for DOX in the literature which can be used in our models to get more accurate results. However, the proposed model can be applied to other drugs by adjusting the drug parameters in the model. Molecular communication is an emerging paradigm for exchange the biochemical molecules between the biological cells and synthetic nanomachines within the biological aqueous environments^25,26^. One of the most important applications of the MC paradigm is modelling and abstraction of the drug delivery systems, particularly for providing the drug at the site of action and minimizing the drug in the healthy tissues^25,26^. In this paradigm, the drug delivery process is abstracted as a communication mechanism, as shown in Fig. 2. The implantable drug delivery device (the implant) acts as a transmitter while the target site, i.e., malignant cell, acts as a receiver. The anticancer therapeutic agent, i.e., DOX, can be considered as information molecules. The tumor microenvironment is a three-dimensional (3-D) medium surrounded by normal tissue, which acts as a molecular communication channel. At the target sites, the intracellular concentration of the anticancer drug (DOX) should reach a minimum threshold to kill the cancer cells. In the MC paradigm, this can be considered as a reception mechanism where the intracellular concentration is the received signal, while the death of cancer cells is the output response.

In this model, a millimeter-scale dual-release implant, loaded with anticancer DOX drug, is assumed to be inserted inside a solid tumor to releases DOX anticancer agents. Here, we consider two solid tumor models, namely, thermally ablated and non-ablated tumors, surrounded by normal tissues. We consider the impact of the following factors on the drug transport process in tumor and surrounding normal tissue: interstitial fluid pressure and velocity, binding of DOX with albumin-proteins in the interstitial extracellular space, cellular influx/efflux of DOX across the cellular membranes, elimination of DOX into the blood and lymphatic microvessels. Impacts of all the above-mentioned pharmacokinetic processes are included in the drug transport model for predicting the extracellular and intracellular concentrations of both free and bound DOX. Furthermore, this model enables estimate the toxicity of DOX on tumor cells and surrounding healthy tissue. The impact of DOX on the cancer cells is evaluated using a pharmacodynamic model that depends on the spatiotemporal intracellular concentration of DOX as well as on the characteristics of both the DOX and tumor cells. Moreover, the concentration of DOX in normal tissue is evaluated, which can be used for toxicity assessment. Accuracy and validity of our proposed model are verified and compared with the published experimental data in the literature, assuming the impact of the various pharmacokinetic parameters are combined in the apparent diffusivity and apparent elimination constant. To the best of our knowledge, this work is the first comprehensive model available in the literature that simultaneously captures and addresses the anticancer drug transport, pharmacokinetics, and pharmacodynamics using local dual-release drug implants in malignant solid tumors following thermal ablation.

## Results and discussion

In this study, the governing mathematical equations which describe the proposed model are discretized in space with the finite element method using the commercial software package COMSOL Multiphysics 5.3. In COMSOL Multiphysics, we solve the interstitial fluid flow and drug transport models together with the tumor cell density model. The steady-state solutions of the interstitial velocity and pressure fields are applied to the drug transport model. Both the fluid flow and the drug transport models are solved under relative tolerance of 10^–6^ and absolute tolerance 10^–7^. The numerical computation is run to examine the model over a timeframe of 4 days (96 h), assuming the initial time is the time of insertion of the implant in the tumor. In this model, the tumor characteristics and the drug parameters are taken from the published experimental works and other studies in the literature. The parameters used in this study are given in Tables 2, 3, 4 and 5.

**Figure 3:**
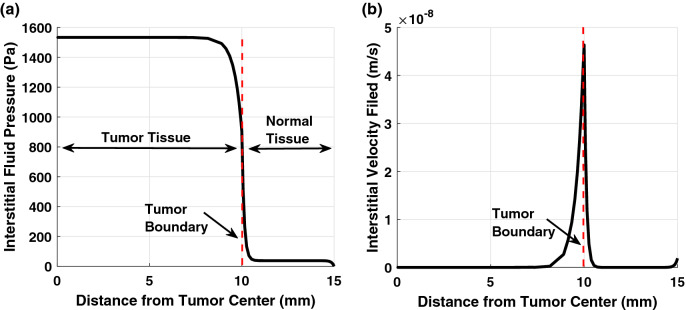
(**a**) Interstitial fluid pressure and (**b**) velocity field in the tumor and normal tissues with the radial distance from the tumor center.

As shown in Fig. 3, the average and maximum interstitial fluid pressure (IFP) in the tumor are equal to 1466 Pa and 1533 Pa, respectively. However, the average pressure in the normal tissue is equal to 50 Pa, which is significantly lower than the pressure in the tumor. Furthermore, there is a sharp pressure gradient (and consequently, high-velocity field) at the interface boundary between the tumor and normal tissues, as shown in Fig. 3. This result is not surprising due to the lake of the lymph vasculature in the tumor compared to the normal tissue. The trend of the results agrees well with the previous studies in the literature^1,27,28^.

**Figure 4:**
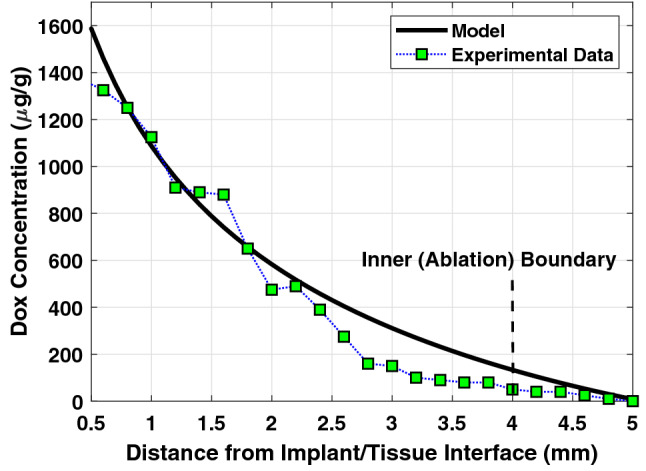
Comparison of DOX concentration obtained from the experimental data^21^ with our models using apparent diffusivity and apparent elimination rate.

A group of researchers conducted experiments for measuring the DOX concentration with the distance from polymer implants placed inside thermally ablated liver tumor^8,20,21,29^. In Fig. 4, we verify the accuracy and validity of our drug transport model by comparing the results with the published experimental data^21^. The impact of the various processes in the tumor, including the binding effect, is given in terms of the apparent elimination rate constant and apparent diffusivity^20^. The parameters used in this comparison are chosen to be the same as that used in the experiment^21^. The apparent diffusivity of DOX in the liver tumor is given as D = 50 µm^2^/s while it varies within the thermally ablated tumor; thus, we use an average value^20^ of 78.2 µm^2^/s. The apparent elimination rate constant of DOX in the liver tumor is γ = 0.58 × 10^–4^ s^−1^, and it is negligible in the ablated tumor within the first 4 days^20^, i.e., γ = 0 s^−1^. As expected, the measured concentration shows a decreasing trend with the radial distance from the implant. The results obtained using our numerical COMSOL model agree well with the results extracted from the published experimental data. This indicates the accuracy and validity of the drug release and transport model, which represents the main part of the proposed model in this paper.

**Figure 5:**
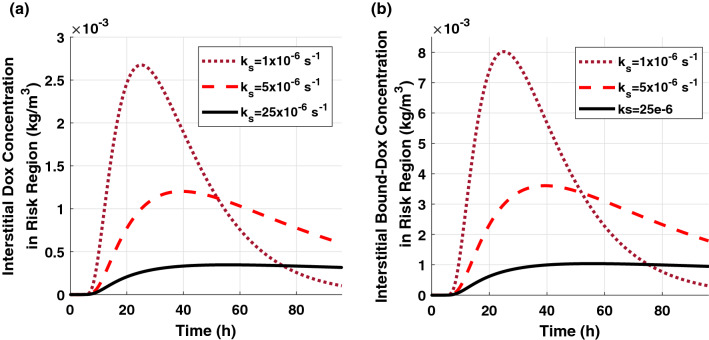
Spatial-mean temporal concentration profile of (**a**) free-DOX and (**b**) bound-DOX in the extracellular space of the risk region in 90% ablated tumor.

In this study, the concentration distribution profiles for both 80% and 90% ablation have a similar trend with the time and distance but with slightly different amplitudes. Therefore, we do not show all the results in this paper to eliminate the redundancy. Figure 5 shows the spatial-mean extracellular concentration profiles of free-DOX and bound-DOX in the risk region of 90% ablated tumor. The implant with a higher release rate leads to a larger peak concentration and lower peak time. As the release rate increases, the amount of the released DOX will increase rapidly, and thus the peak concentration will reach a higher value within a shorter time. The increasing and decreasing rates of the extracellular concentration become sharper as the release rate constant increases. The decay in concentration after it reaches the peak value is due to a reduction in the released drug from the implant and elimination through blood vessels and cellular uptake. The bound-DOX concentration has a similar trend as the free-DOX concentration for various sustained release rate constants but with approximately three-fold higher amplitude.

**Figure 6:**
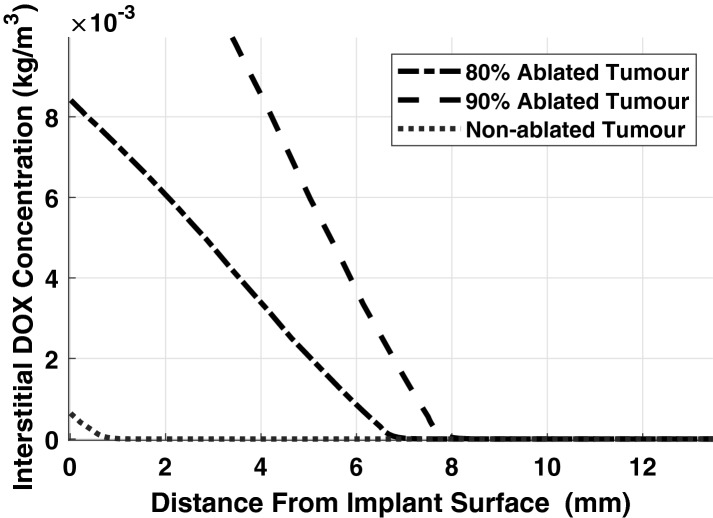
Spatial extracellular concentration of free-DOX at t = 96 h (fourth day), after insertion of the implant in a solid tumor with/without RFA.

Figure 6 shows the extracellular free-DOX concentration versus the distance from the implant/tissue interface in ablated and non-ablated solid tumors. The extracellular DOX concentration in a solid tumor without applying thermal ablation has a smaller amplitude than the concentration in an ablated tumor. This happens because in the non-ablated tumor, the cells and blood vasculature structures, which cover a large volume of the solid tumor, have a high impact on the elimination of the DOX through the cellular uptake and the blood microvessels. Furthermore, the drug can penetrate a larger distance and cover a larger volume in the ablated tumor compared to the non-ablated tumor. Also, tumor with larger ablation radius (e.g., 90%) shows higher DOX concentration and larger penetration compared to smaller ablation radius (e.g., 80%) on the fourth day as shown in Fig. 6. The observed impact of the thermal ablation on the drug distribution in tumors agrees with the experimental data in the literature^6,8,21^.

**Figure 7:**
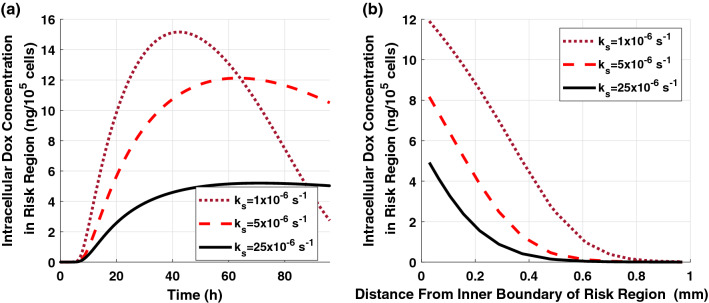
Intracellular free-DOX concentration in the risk region of 90% ablated tumor for various release rate constants (**a**) spatial-mean temporal concentration profile and (**b**) spatial concentration at t = 12 h.

The intracellular concentration of free-DOX in the risk region of 90% ablated tumor is shown in Fig. 7. The DOX intracellular concentration follows a similar trend as the extracellular concentration because it highly depends on the extracellular DOX levels. The higher peak amplitude of the intracellular concentration can be achieved using a faster release implant. As shown in Fig. 7b, the intracellular concentration decreases as the distance increases from the inner boundary of the risk region. This happens because the extracellular DOX concentration decreases with the distance, and it has a direct influence on the intracellular uptake and, consequently, on the intracellular DOX concentration.

**Figure 8:**
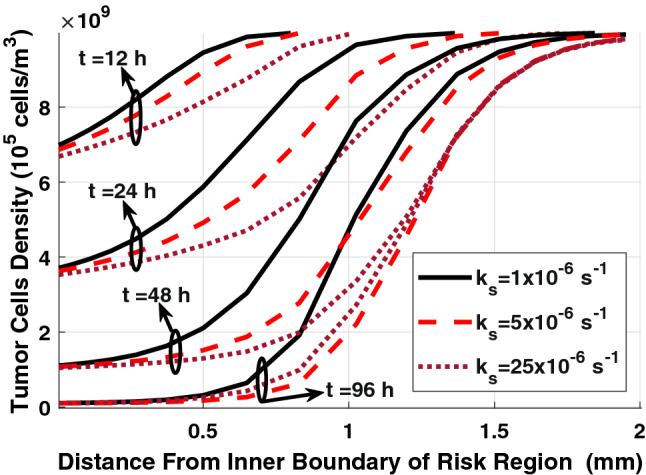
Spatial distribution of tumor cell density in the risk region of 80% ablated tumor for various sustained release rate constants.

Figure 8 shows the tumor cell density in the risk region with the radial distance from the inner boundary of the risk region of 80% ablated tumors at various times following the insertion of the implant. Tumor cell density shows heterogeneous distribution along the radial direction, where it increases with the distance with minimum density appears near the inner boundary of the risk region. This can also be seen in the color map of the spatial distribution of tumor cell density in Fig. 10. Moreover, there is a significant reduction in tumor cell density over time following the insertion of the implant. Furthermore, we found that the implant with the release rate constants k_s_ = {5, 25} × 10^–6^ s^−1^ will almost have the same therapeutic effect on the last day of treatment. However, using a smaller release rate will consume less amount of the drug with minimum toxicity on the normal tissue. Therefore, design the implant with an optimal release rate is very important to get a high therapeutic efficacy.

**Figure 9:**
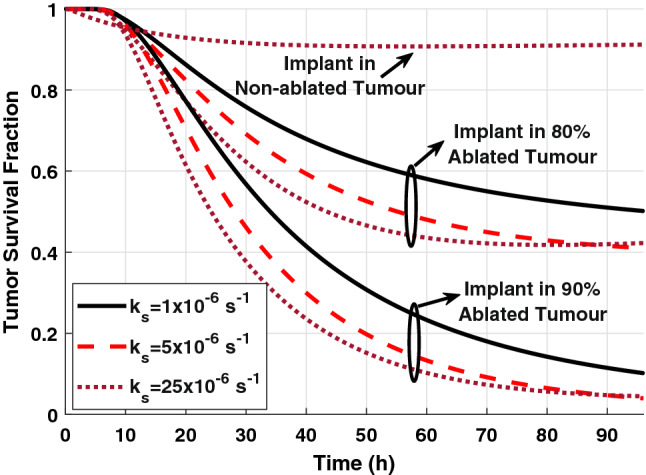
The survival fraction of tumor cells in non-ablated and ablated tumors for various sustained release rate constants.

**Figure 10:**
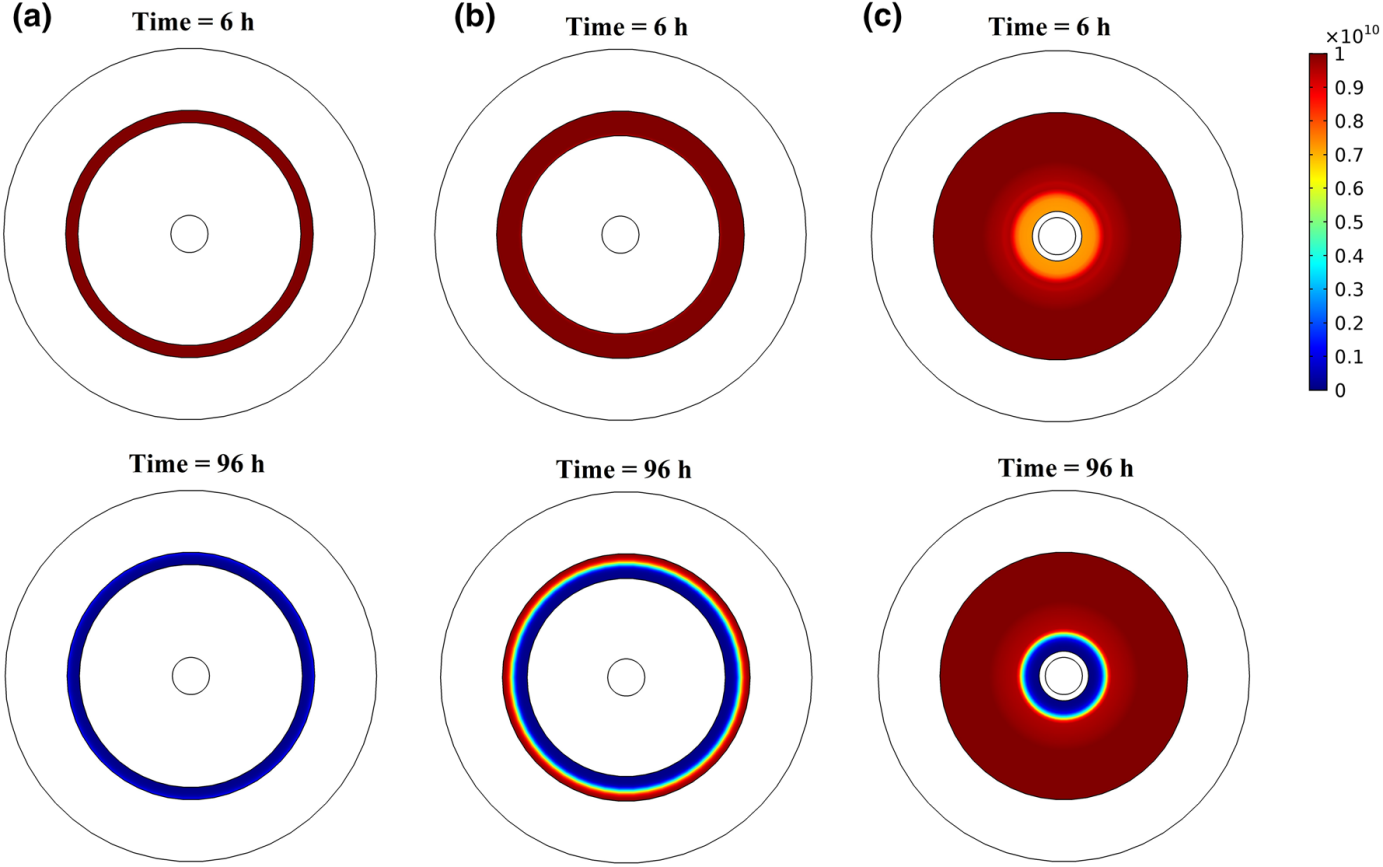
Cross-sectional view of the spatial distribution of tumor cell density in the risk region at different times for (**a**) 90% ablated tumor, (**b**) 80% ablated tumor, and (**c**) tumor without RFA when *k*_*s*_ = 5 × 10^−6^ s^−1^.

The final therapeutic outcomes of the combination therapy using the DOX-loaded implant and RFA can be obtained from the tumor survival curves, as shown in Fig. 9. We can see that using the implant alone for tumor treatment without RFA leads to a negligible impact on the tumor cell density, even with a high release rate, i.e., 90% of the tumor cells survive at the end of the treatment (at t = 96 h). In the case of 80% ablated tumor, there is about 40% cancer cells survive on the last day of the treatment. However, for 90% ablated tumor, most of the tumor cells are killed on the last day, i.e., only 4% survival cells. This can also be confirmed in the color map of tumor cell distribution, as shown in Fig. 10. Thus, the combination treatment using the implant following RFA will result in high therapeutic efficacy in destroying the residual tumor cells compared to a single therapy approach. Moreover, the release rate constants, k_s_ = {5, 25} × 10^−6^ s^−1^, show a similar therapeutic effect on the last day (at t = 96 h) with lower tumor survival compared to an implant with k_s_ = 1 × 10^−6^ s^−1^.

In general, the IDDSs have negligible toxicity on the surrounding healthy tissue and do not cause systemic toxicity. However, the amount of DOX which may reach to the normal tissue should be minimized to reduce the toxicity risks. The DOX level in the normal tissue can be used as a metric to predict the toxicity. In this study, the peak DOX concentration in normal tissue under all the examined release rates, as listed in Table 1, is found to be lower than the half-maximal inhibitory concentration^27^ (IC_50_ = 4.13 × 10^−5^ kg/m^3^). The peak extracellular concentration of free and bound-DOX in the solid tumor without RFA has very small and negligible values. In the non-ablated solid tumor, the released DOX from the implant will be affected by the cellular uptake and the elimination through the blood vessels before reaching the normal tissue. This explains why the amount of DOX that appears in the normal tissue is very small in the case of the non-ablated tumor compared to the ablated tumor where the vascular and cellular structures are destroyed.

**Table 1:** The maximum DOX concentration in the normal tissue for ablated and non-ablated tumors.

Tumor	Drug	Release rate constant		
		*k*_*s*_ = 1 × 10^–6^ s^−1^	*k*_*s*_ = 5 × 10^–6^ s^−1^	*k*_*s*_ = 25 × 10^–6^ s^−1^
90% ablation	Free	58.9 × 10^–8^	202 × 10^–8^	430 × 10^–8^
	Bound	176 × 10^–8^	608 × 10^–8^	1280 × 10^–8^
80% ablation	Free	0.28 × 10^–8^	0.99 × 10^–8^	2.04 × 10^–8^
	Bound	0.85 × 10^–8^	2.98 × 10^–8^	6.13 × 10^–8^
No ablation	Free	0.72 × 10^–12^	0.74 × 10^–12^	1.10 × 10^–12^
	Bound	2.16 × 10^–12^	2.21 × 10^–12^	3.32 × 10^–12^

## Conclusions

In this paper, we propose comprehensive mathematical and computational models for the transport of anticancer drug following the insertion of a dual-release implant in thermally ablated solid tumor with the help of the molecular communication paradigm. We predict the extracellular and intracellular concentrations of both free and bound-DOX in the various regions of thermally ablated solid tumor. This model includes the impact of the various pharmacokinetic processes such as binding of DOX to proteins, cellular influx/efflux, and elimination into the vasculature system. Also, we investigate the impact of the pressure and velocity of interstitial fluid on DOX transport in tumor and surrounding healthy tissue. Accuracy and validity of the proposed transport model is verified with the published experimental data assuming that the various pharmacokinetic processes are combined in the apparent diffusivity and elimination constant. Moreover, we examine the impact of the anticancer drug on tumor cell density, which shows a significant reduction in cell density over time. The combination therapy using the implantable drug delivery following thermal ablation results in high therapeutic efficacy. We found that the anticancer drug does not lead to toxicity effect on the normal tissue. The proposed model can help to optimize the development of the combination technique for treating solid tumors. Thus, we can reduce the clinical trials and the number of animals in biomedical research to save time and reduce cost. One of the limitations in our model is ignoring the impact of the implant biodegradation and drug interactions with the tissue surrounding the implant on the drug release process. Another limitation is using average diffusivity within the ablated tumor. However, the diffusivity varies with the radial distance in the ablated tumor. We will improve our model to overcome these limitations in future works.

## Method

### Mathematical model

**Figure 11:**
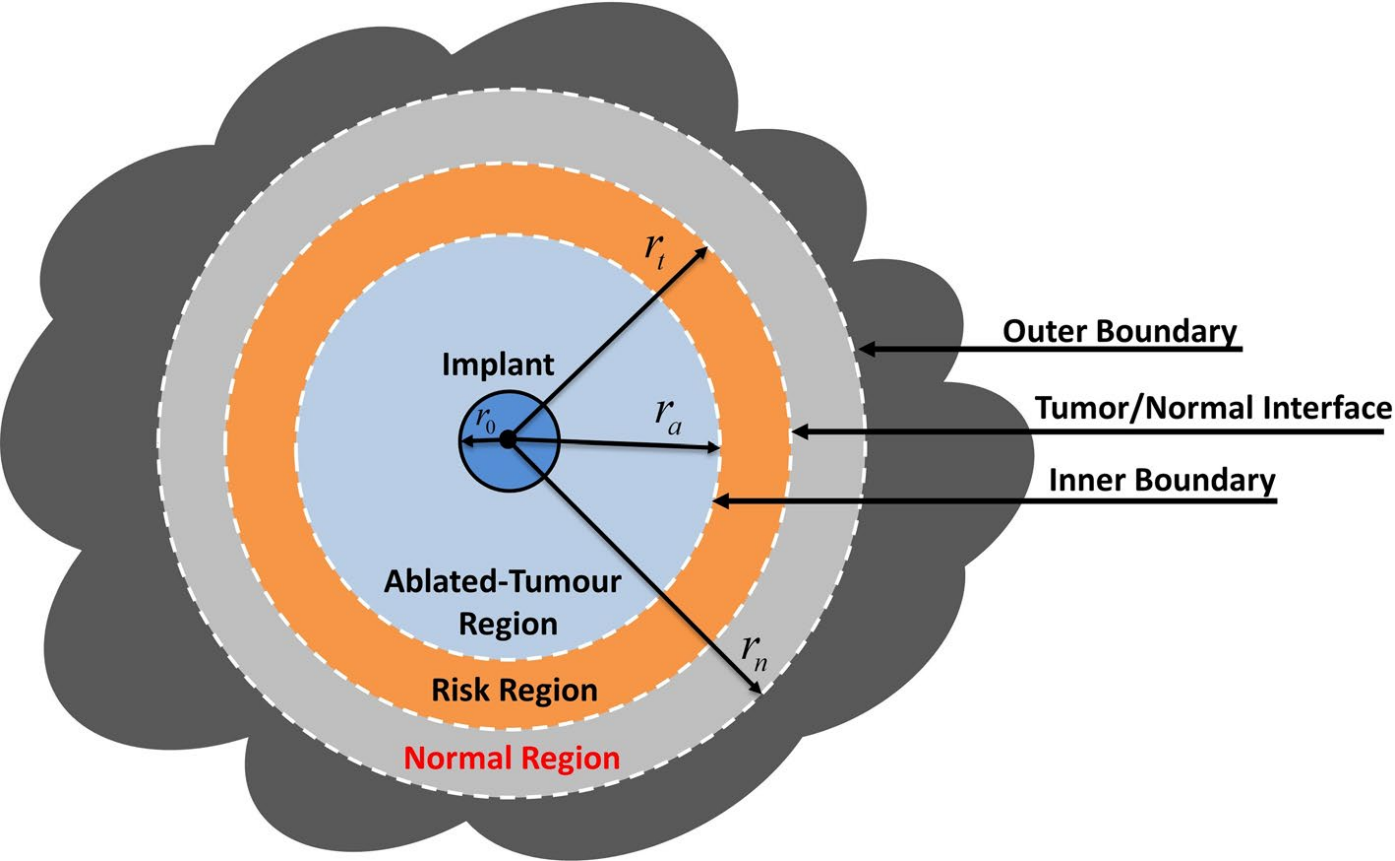
Schematic illustration of a cross-section view of a 3-D solid tumor, including the drug implant after RFA.

Solid tumors are heterogeneous environments due to spatial heterogeneity of the tumor vasculature and the cells. However, due to the unavailability of experimental heterogeneity data of solid tumors and to simplify the analysis, the solid tumors are widely treated in the literature as spatially homogeneous media^27,28,30–32^. Thus, we do not discriminate between the necrotic and viable tumor regions. Moreover, we assume that the growth timescale of the tumor and normal tissues is much longer than the timescale of the transport phenomena and the observation time window. Thus, it would be reasonable to assume that the system's physiological parameters to be time-independent^27^. Incomplete radiofrequency ablation (RFA) will create an ablated zone, with no viable cancer cells, surrounded by a tumor rim (risk region) that shows a high density of the viable malignant cells, as shown in Fig. 11.

### Tumor microenvironment modelling

#### Interstitial fluid transport

The tumor and surrounding tissue can be treated as porous media because the length scale of the intercapillary distances is much smaller than the tumor radius^27,30,33,34^. Thus, the variations over the microscopic length scales can be averaged out, and the interstitial fluid flow (IFF) is defined by coupling mass and momentum conservation equations.

For incompressible Newtonian fluid flow through a porous medium, the momentum conservation equation (Navier–Stokes equation) is simplified to Darcy’s law at a steady-state, which is quite applicable to the analysis of the interstitial fluid flow^33^. Darcy's law is used to account for the convective contribution of the interstitial fluid through porous media. Darcy law is derived for incompressible Newtonian fluid with neglecting the following factors: the divergence of the velocity, the inertial force, and the friction within the fluid and between the fluid and solid phases. Similar to other works in the literature^35,36^, the fluid flow in the tumor tissue with thermal ablation can be modelled using Darcy law.

1 $$ \vec{v}_{i} = - k_{i} \nabla P_{i} $$

where $\vec{v}_{i}$ is the interstitial velocity field (IVF) in (m/s), $P_{i}$ is the interstitial fluid pressure (IFP) in (Pa), $k_{i}$ is the hydraulic conductivity of the interstitial fluid in ${\text{(m}}^{{2}} {\text{/(Pa}}\;{\text{s))}}$.

The mass continuity (balance) equation for an incompressible fluid in the porous media with source and sink of mass is given as follows^27^.

2 $$ \frac{{\partial \rho_{i} }}{\partial t} + \nabla \cdot \left( {\rho_{i} \vec{v}_{i} } \right) = \left( {\phi_{vi} - \phi_{li} } \right)\rho_{i} $$

where $\rho_{i}$ is the interstitial fluid density in (kg/m^3^), $\phi_{vi}$ is the mass fluid source term which represents the fluid flow rate per unit volume of tissue from the blood vessels into the interstitial space in (1/s), and $\phi_{li}$ is the lymphatic drainage (sink) term that represents the fluid flow rate per unit volume of tissue from the interstitial space into the lymph vessels in (1/s). The Eq. (2) is applicable to both normal and cancerous biological tissues.

The mass fluid source term $\phi_{vi}$ is governed by Starling’s law^27,30^ as

3 $$ \phi_{vi} = \frac{{L_{vi} S_{vi} }}{{V_{i} }}\left( {P_{vi} - P_{i} - \sigma_{i} \left( {\pi_{vi} - \pi_{i} } \right)} \right) $$

where $P_{vi}$ is the intervascular blood pressure (IBP) in (Pa), $\pi_{vi}$ is the osmotic pressure of the plasma in (Pa), $\pi_{i}$ is the osmotic pressure of the interstitial fluid in (Pa), $L_{vi}$ is the hydraulic conductivity of the blood vessel walls in (m/Pa s), $\sigma_{i}$ is the average osmotic reflection coefficient for plasma proteins, and ${{S_{vi} } \mathord{\left/ {\vphantom {{S_{vi} } {V_{i} }}} \right. \kern-\nulldelimiterspace} {V_{i} }}$ is the surface area of the blood vessels per unit volume of tissue in (1/m).

The lymphatic drainage term $\phi_{li}$ is given as^27,30^

4 $$ \phi_{li} = \frac{{L_{li} S_{li} }}{{V_{i} }}\left( {P_{i} - P_{li} } \right) $$

where $P_{li}$ is the hydrostatic pressure of intra-lymphatic in (Pa), $L_{l}$ is the hydraulic conductivity of the lymphatic wall in (m/(Pa s)), ${{S_{li} } \mathord{\left/ {\vphantom {{S_{li} } {V_{i} }}} \right. \kern-\nulldelimiterspace} {V_{i} }}$ is the surface area of the lymphatic vessels per unit volume of tissue in (1/m), and ${{L_{l} S_{l} } \mathord{\left/ {\vphantom {{L_{l} S_{l} } V}} \right. \kern-\nulldelimiterspace} V}$ is the lymphatic filtration coefficient in (1/(Pa s)).

At steady state, the mass continuity equation for incompressible flow in porous media reduces to

5 $$ \nabla \cdot \vec{v}_{i} = \phi_{vi} - \phi_{li} $$

Now, by combining Darcy’s law (1) and the mass continuity Eq. (5), we get

6 $$ - k_{i} \nabla^{2} P_{i} = \phi_{vi} - \phi_{li} $$

where $\nabla^{2}$ is the Laplacian operator.

The lymphatic drainage term is equal to zero in the tumor region due to the lake of the lymphatic system. Moreover, the mass fluid source term is equal to zero in the ablated-tumor region because the thermal ablation destroys the vascular network. Thus, Eq. (6) can be rewritten as

7 $$ - k_{i} \nabla^{2} P_{i} = \left\{ {\begin{array}{*{20}l} 0, \hfill \\ {\phi_{vi} }, \hfill \\ {\phi_{vi} - \phi_{li} }, \hfill \\ \end{array} } \right.\begin{array}{*{20}c} {\text{Ablated tumour }} \\ {\text{ Non - ablated tumour}} \\ {\text{Normal tissue }} \\ \end{array} $$

Equation (7), together with the boundary conditions (23)–(34), can be solved analytically or numerically. However, the analytical derivation could be complicated for three regions even there is an analytical solution for two regions model, and thus we solve it using COMSOL Multiphysics software. The obtained interstitial fluid pressure and velocity will be used in the drug transport model in the next subsection.

#### Interstitial drug transport

In this model, the drug source is a miniaturized implant loaded with DOX anticancer drug inserted inside a solid tumor. The implant will release the anticancer drug, which diffuses through the surrounding tissue, and it will be influenced by the various pharmacokinetic processes, including drug efflux/influx into cells, drug binding/unbinding with interstitial proteins, elimination into blood capillaries, and biodegradation. These factors have a significant impact on drug transport and therapeutic efficacy.

Transport of free-DOX in the interstitial space can be mathematically modelled using the following diffusion–convection–reaction equation.

8 $$ \frac{{\partial C_{exi} }}{\partial t} + \nabla \cdot \left( {v_{i} C_{exi} } \right) = \nabla \cdot \left( {D_{Fi} \nabla C_{exi} } \right) + F_{Bi} + F_{Ci} - \gamma_{i} C_{exi} $$

where $\nabla$ is the Del gradient operator and the index $i \in \left\{ {a,t,n} \right\}$ refers to the ablated tumor, the non-ablated tumor, and the normal tissues. The function $C_{exi}$ is the spatiotemporal extracellular concentration of free-DOX in the interstitial space in (g/m^3^), and $D_{Fi}$ is the diffusion coefficient of free-DOX, which has a different value in each region.

The loss rate of free-DOX due to drainage in the lymphatic vessels and elimination by the blood capillaries.

9 $$ \gamma_{i} = \left\{ {\begin{array}{*{20}l} 0, \hfill \\ {\gamma_{vi}}, \hfill \\ {\gamma_{vi} + \phi_{li} } ,\hfill \\ \end{array} } \right.\begin{array}{l} {\text{ Ablated Tumor }} \\ {\text{ Non - ablated Tumor}} \\ {\text{ Normal Tissue }} \\ \end{array} $$

The loss rate of free-DOX due to lymphatic drainage $(\phi_{li} )$ is given by Eq. (4). Since lack of lymphatic system in the tumor region^1,28,30^, we set the loss rate due to lymphatic drainage equal to zero in both the ablated and non-ablated tumor regions. Moreover, the experimental studies showed that thermal ablation destroys the vascular structure inside the ablated tumor zone, and therefore the DOX loss into the blood in this region is neglected. However, several blood microvessels may appear in the peripheral region of the ablation zone^37^. Moreover, the initial concentration of DOX in the plasma is assumed to be zero, where the implant is the only drug source^38^.

The loss rate of free-DOX due to elimination by blood vessels $(\gamma_{vi} )$ can be expressed as follows^38,39^.

10 $$ \gamma_{vi} = \frac{{P_{Fi} S_{vi} }}{{V_{i} }} $$

where $P_{Fi}$ is the permeability coefficient of the blood vessel wall to free-DOX in (m/s).

The term $F_{B}$ accounts for the binding and unbinding of DOX with Albumin proteins in the interstitial space.

11 $$ F_{Bi} = k_{d} C_{bi} - k_{a} C_{exi} $$

where $k_{a}$ and $k_{d}$ are the DOX-protein association and dissociation reaction rates, respectively, and $C_{bi}$ is the spatiotemporal bound-DOX concertation in the tissues.

The doxorubicin molecules can transport to/from the interior of the cell across the cell membrane. Thus, the effect of cellular uptake (influx) and efflux is modelled using the following cellular influx/efflux rate:

12 $$ F_{Ci} = D_{c} \left( {\zeta_{eff} - \zeta_{\inf } } \right) $$

where $D_{c}$ is the cancer cells density in the unit of (10^5^ cells/m^3^). The cellular influx and efflux functions, $\zeta_{\inf }$ and $\zeta_{eff}$, are given in Eqs. (14) and (15), respectively.

### Modelling of the target cells

#### Drug absorption by the cells

The doxorubicin can transport across the cell membrane via passive diffusion and carrier-mediated transport^40^. Free and bound DOX can cross the tumor cell membrane. The amount of bound DOX which enter the cells is neglected^41^. Therefore, we assume that only free-DOX can uptake by the cells, and therefore the intracellular concentration is a function of the extracellular free-DOX concentration. The intracellular DOX concentration $C_{c}$ is expressed in the unit of (ng/10^5^ cells), and it changes with the time according to the following equations^42,43^.

13 $$ \frac{{dC_{c} }}{dt} = \zeta_{\inf } - \zeta_{eff} $$

14 $$ \zeta_{\inf } = V_{\max } \frac{{C_{exi} }}{{C_{exi} + k_{e} \phi_{e} }} $$

15 $$ \zeta_{eff} = V_{\max } \frac{{C_{c} }}{{C_{c} + k_{c} }} $$

where $\zeta_{\inf }$ and $\zeta_{eff}$ are the cellular influx and efflux functions, $V_{\max }$ is the maximum rate of transmembrane transport, and $\phi_{e}$ is the extracellular volume fraction. The parameters $V_{\max }$, $k_{e}$, and $k_{c}$ were obtained in the literature^43^, by the fitting of the experimental data of intracellular Adriamycin concentration in tumor cells^44^. The parameters mentioned above are listed in Table 2.

**Table 2:** Tumor cell density and intracellular concentration parameters.

Parameter	Unit	Value	References
$V_{\max }$	ng/(s 10^5^ cells)	4.67 × 10^−3^	^27,41–43,45,46^
$k_{e}$	kg/m^3^	2.19 × 10^−4^	^27,41–43,45,46^
$k_{c}$	ng/10^5^ cells	1.37	^27,41–43,45,46^
$\phi_{e}$	–	0.4	^27,41,43,45^
$D_{c0}$	10^5^ cells/m^3^	1 × 10^10^	^27,41,42,45,46^
$k_{gr}$	s^−1^	5.78 × 10^−6^	^42,46^
$k_{dr}$	s^−1^	2.78 × 10^−6^	^42,46^
$k_{m}$	m^3^/(s 10^5^ cells)	3 × 10^−16^	^27,42,46^
$k_{\max }$	s^−1^	1.67 × 10^−5^	^27,47^
$EC_{50}$	ng/10^5^ cells	0.5	^27,47^

Albumin is moving protein molecules that can be found in the bloodstream and interstitial space. Some free-DOX molecules may bind to proteins, such as Albumin, in the interstitial space, then new macromolecules (bound-DOX) will be created. The bound-DOX may unbind from the DOX-protein complexes and then becomes free. The spatiotemporal transport of bound-DOX in the extracellular space can be modelled using the following diffusion–convection–reaction equation^27,41^:

16 $$ \frac{{\partial C_{bi} }}{\partial t} + \nabla \cdot \left( {v_{i} C_{bi} } \right) = \nabla \cdot \left( {D_{Bi} \nabla C_{bi} } \right) - F_{Bi} - \gamma_{i} C_{exi} $$

where $C_{exi}$ is the spatiotemporal extracellular concentration of bound-DOX in the interstitial space and $D_{Bi}$ is the diffusion coefficient of bound-DOX, which has a different value in each region.

The loss rate of bound-DOX due to the blood and lymphatic microvessels is given as

17 $$ \gamma_{vi} = \frac{{P_{Bi} S_{vi} }}{{V_{i} }} $$

where $P_{Bi}$ is the permeability coefficient of the blood vessel wall to bound-DOX.

#### Death of the tumor cells

The cancer cell density can be described using the following equations, which include the impact of the free-DOX concentration and the natural growth and death of cells^27,42^.

18 $$ \frac{{dD_{c} }}{dt} = \left( {k_{gr} - k_{dr} } \right)D_{c} - KD_{c} - k_{m} D_{c}^{2} $$

19 $$ K = \frac{{k_{\max } C_{c} }}{{C_{c} + EC_{50} }} $$

where $k_{gr}$ and $k_{dr}$ are the natural growth and decay rate constants of the tumor cells, respectively, and $k_{m}$ is the saturation constant. The nonlinear function K reflects the effect of the anticancer drug, which depends on the intracellular concentration of free-DOX, the maximal DOX cell killing rate ($k_{\max }$), and Michaelis constant $EC_{50}$. Initial tumor cell density is used as an initial condition for solving Eq. (18). The parameters mentioned above are listed in Table 2.

### Drug implant model

In this study, the drug implant is loaded with anticancer drug DOX. The main design parameters of the implant are the release rate, the implant size, and the amount of loaded drug. The release rate depends on the implant formation and the physicochemical properties of the loaded drug, which can be adjusted during the design phase by selecting appropriate materials, e.g., polymer and drugs^48^. The optimal release rate can help in improving the therapeutic outcomes by reducing the side effects, which in order save time and cost. The amount of the released drug can be experimentally monitored over time to obtain the release profiles. Relevant mathematical models for the release kinetic can be fitted to the experimentally measured release curves to predict the release rate constants^49^. The implant has a dual-release pattern, i.e., it releases DOX over two phases: fast burst release over a short time duration followed by slow sustained release over an extended period of time. Thus, we can model both dual-release and sustained release implants by adjusting the release parameters. For example, in-situ forming implants (ISFIs) and double-layer implants have dual-release pattern^6,12^.

The cumulative amount of released DOX at the time *t* can be mathematically modelled using the bi-exponential first-order kinetic model as

20 $$ M\left( t \right) = M_{0} W_{\infty } \left( {1 - f \cdot e^{{ - k_{f} t}} - \left( {1 - f} \right) \cdot e^{{ - k_{s} t}} } \right) $$

where $M_{0}$ is the total amount of loaded-drug in the implant in (mg), *W*_∞_ is the total fraction of drug released at steady state, and *f* is a fraction of drug released during the burst phase. The parameters $k_{f}$ and $k_{s}$ are release rate constants for burst and sustained release phases in (s^−1^), respectively.

Now, the rate of drug release across the spherical implant surface at time *t* per unit area of the implant surface in g/(s m^2^), i.e., the flux, can be expressed as

21 $$ F_{rs} \left( t \right) = \frac{1}{A}\frac{dM}{{dt}} = \frac{{M_{0} W_{\infty } }}{A}\left( {f \cdot k_{f} e^{{ - k_{f} t}} + \left( {1 - f} \right) \cdot k_{s} e^{{ - k_{s} t}} } \right) $$

where $A = 4\pi r_{0}^{2}$ is the surface area of the implant and *r*_0_ is the implant radius.

The characteristic parameters of the dual-release implant are listed in Table 3. Similar to the experimental release data^6^, we chose 10% of the loaded drug to be released within the first hour while different values of sustained-release rate constant are examined.

**Table 3:** The dual-release implant parameters.

Parameter	Value	Unit
*M*_0_	5	mg
*k*_*f*_	5 × 10^–4^	s^−1^
*k*_*s*_	1–25 × 10^–6^	s^−1^
*f*	0.1	–
*W*_*∞*_	1	–
*r*_0_	1.5	mm

The experimental studies in the literature showed insignificant variation for the size of the implant during the release duration since the implant releases drug prior to any significant degradation^38^. Thus, it is reasonable to assume that the size variation due to biodegradation is negligible during the observation time^6,38^.

### Model parametrization

In this work, the values of the model parameters are obtained from the published experimental data and other studies in the literature, assuming that the growth of the tissues is negligible during the observation timeframe. This assumption is widely used in the literature since the time scales of fluid and drug transport phenomena are relatively short^27^. Thus, it would be reasonable to assume that the system's physiological parameters to be time-independent. The parameters are defined through the paper and summarized in Tables 2, 3, 4 and 5.

#### Microvasculature density (S/V)

The microvasculature density is the ratio of vascular surface area per unit volume of tissue. The microvasculature density of capillaries highly varies among tumor types and within the same type of tumor^34^. However, the experimental data show a leak of lymph vessels in the tumor tissues. Different studies show that the larger tumors have smaller microvasculature densities^34,50^. For a tumor with 2 cm diameter (i.e., volume = 4188 mm^3^), the surface area of the blood vessels per unit volume of tumor tissue is approximately equal to 10^4^ 1/m^34^. In normal tissues, the surface area of the blood vessels per unit volume of tissue is measured as 7 × 10^3^ 1/m^51^. As mentioned before, the histological analysis confirms that the thermal ablation destroys the vascular network and tumor cells in ablated tumor tissue^4,8,12,21,52–54^. Thus, the effect of microvasculature density and, consequently, drug loss through blood microvessels in ablated tumor tissue is neglected.

**Table 4:** Fluid transport parameters.

Parameter	Unit	Tumor tissue	Normal tissue	References
$\pi_{i}$	Pa	2000	1333	^27,28,30,32,55–57^
$\pi_{vi}$	Pa	2666	2666	^27,28,30,32,55–57^
$\rho_{i}$	kg/m^3^	1000	1000	^27,32^
$\mu_{i}$	Pa s	7.8 × 10^−4^	7.8 × 10^−4^	^27,32^
$k_{i}$	m^2^/(Pa s)	3.10 × 10^−14^	6.40 × 10^−15^	^27,28,30,32,55–57^
${{S_{vi} } \mathord{\left/ {\vphantom {{S_{vi} } {V_{i} }}} \right. \kern-\nulldelimiterspace} {V_{i} }}$	m^−1^	10,000	7000	^27,28,30,32,34,51,55–58^
$L_{vi}$	m/(Pa s)	2.10 × 10^−11^	2.70 × 10^−12^	^27,28,30,32,55–57^
${{L_{l} S_{l} } \mathord{\left/ {\vphantom {{L_{l} S_{l} } V}} \right. \kern-\nulldelimiterspace} V}$	1/(Pa s)	0	4.17 × 10^−7^	^27,30^
$P_{li}$	Pa	0	0	^27,30^
$P_{vi}$	Pa	2080	2080	^27,28,30,32,55–57^
$\sigma_{i}$	–	0.82	0.91	^27,28,30,32,55–57^

#### Extracellular space fraction $(\phi_{e} )$

The volume fraction of extracellular space in the tumor is much larger than that in the normal tissue, and it ranges from 0.2 to 0.6 with tumor cell density ranges from 0.955–15.3 10^5^ cells/mm^3^^41^. In this study, the volume fraction of extracellular space $\phi_{e}$ and the initial cell density $D_{c0}$ are chosen to be equal to 0.4 and 10 × 10^5^ cells/mm^3^, respectively.

#### Microvasculature permeability (P_F_, P_B_)

The vasculature permeability measures the capability of the blood or lymph microvessels to exchange various substances in and out of the vasculature. The vasculature permeability of Albumin (similar to Albumin bound-DOX) and free-DOX is approximately threefold higher in tumor than that in normal tissues^41^.

#### Diffusion coefficients (D_F_, D_B_)

The diffusivity of free-DOX is higher than Albumin-bound DOX, where the molecular weights (MW) of free-DOX and bound-DOX are 544 Da and 69 kDa, respectively. Moreover, the diffusivity of free and bound-DOX in the tumor is larger than that in normal tissues, as listed in Table 5. However, the experimental studies found that the diffusivity of DOX near the center of the ablated liver tumor after RFA, $D_{ac}$, is 75% higher than that in non-ablated tumor^20,23^. Based on the histological findings in ablated tumor tissues, the diffusivity shows dependency on the radial distance with a higher value at the center of the ablated tumor than the periphery region. The diffusion coefficient in the outer region of the ablated tumor ($r_{c} \le r \le r_{a}$) is characterized as^20,23^

22 $$ D_{a} = D_{ac} - \frac{{r - r_{c} }}{{r_{a} - r_{c} }}\left( {D_{ac} - D_{t} } \right) $$

where *r*_*a*_ is the ablation zone radius, $r_{c} = \alpha r_{a}$ is the radius of the central region of the tumor, and $\alpha = 0.47$.

The diffusivity $D_{ac}$ of the tumor center decreases linearly with the radial distance to finally reaches the diffusivity of the non-ablated (risk) tumor, i.e., $D_{t}$. In this model, we use an average diffusion coefficient within the ablated liver tumor tissue. The mean diffusivity of the outer ablated region is calculated using the scale relationship of DOX diffusivity in ablated and non-ablated tissues, then by taking the average of the diffusivity given by Eq. (22).

### Model geometry

In this work, a 3-D spherical solid tumor is considered with a diameter of 2 cm, as shown in Fig. 11. This value falls within the range of different tumor sizes encountered in reality^59^, e.g., the diameter of tumors in rats and rabbits ranges from 0.5 to 2 cm. We examine three cases for the tumor microenvironment, namely, non-ablated tumor, 80% ablated tumor, and 90% ablated tumor. For example, 80% ablated tumor means that a tumor region of a radius ($0.8 \times r_{t}$) is ablated. In the case of the ablated tumor, a thin rim of viable cancer cells (risk region) will remain at the tumor periphery. The thickness of the risk regions with 80% and 90% ablation zones are 2 mm and 1 mm, respectively. Here, the thickness of the normal region is chosen to be 5 mm. The implant has a radius of 1.5 mm, and it is located at the tumor center. The model geometry and mesh are created using a built-in CAD kernel in COMSOL Multiphysics package. The mesh size is chosen as “Finer” based on a convergence mesh independence test, which shows that a 5-times decrease in the mesh element size will provide a negligible enhancement, i.e., < 5%, in DOX concentration profiles. Moreover, we refined the mesh using “Boundary Layer Setting” at the implant/tissue boundary and other interface boundaries between the various regions, to handle the rapid change of drug concentration, velocity, and pressure at these interface layers.

**Table 5:** Free and bound doxorubicin parameters.

Parameter	Unit	Free	Bound	References
*D*_*a*_	m^2^/s	5.34 × 10^−10^	13.95 × 10^−12^	Calculated according to the experimental data^20,23^
*D*_*t*_	m^2^/s	3.40 × 10^−10^	8.89 × 10^−12^	^27,32,41–43,45,46,60–62^
*D*_*n*_	m^2^/s	1.58 × 10^−10^	4.17 × 10^−12^	^27,32,41–43,45,46,60–62^
$k_{a}$	s^−1^	0.833	–	^27,41,42,45,46^
$k_{d}$	s^−1^	–	0.278	^27,41,42,45,46^
*P*_*t*_	m/s	1 × 10^−6^	7.8 × 10^−9^	^27,41,43,63^
*P*_*n*_	m/s	3.33 × 10^−7^	2.6 × 10^−9^	^27,41,43,63^
*MW*	kg/mol	0.544	69	^27,32,43,45^

### Boundary conditions

Due to spherical symmetry, the pressure at the tumor center is characterized using no-flux boundary condition as

23 $$ \left. {\nabla P_{i} } \right|_{r = 0} = 0. $$

In this work, the observation time scale in the numerical analysis is assumed to be much shorter than the time scale for the growth of the tumor and normal tissues^27^. Therefore, the interface boundary between the various regions are assumed to be fixed. The continuity boundary conditions of the interstitial pressure and fluid flux are imposed at these interface boundaries as

24 $$ \left. {P_{i} } \right|_{{r = r_{a}^{ - } }} = \left. {P_{i} } \right|_{{r = r_{a}^{ + } }} $$

25 $$ \left. {P_{i} } \right|_{{r = r_{t}^{ - } }} = \left. {P_{i} } \right|_{{r = r_{t}^{ + } }} $$

26 $$ - k_{a} \left. {\nabla P_{i} } \right|_{{r = r_{a}^{ - } }} = - k_{t} \left. {\nabla P_{i} } \right|_{{r = r_{a}^{ + } }} $$

27 $$ - k_{t} \left. {\nabla P_{i} } \right|_{{r = r_{t}^{ - } }} = - k_{n} \left. {\nabla P_{i} } \right|_{{r = r_{t}^{ + } }} $$

In addition, the outer boundary of the normal region is assumed to be fixed. The interstitial fluid pressure at this boundary has a minimal constant value. Thus, Dirichlet boundary condition can be applied at the outer boundary as

28 $$ \left. {P_{i} } \right|_{{r = r_{n} }} = P_{\infty } $$

The total interstitial drug flux is a combination of diffusion and convection fluxes. In this model, the interstitial velocity field mainly appears at the interface boundary between the tumor and the normal tissues due to a large pressure difference at that layer. The continuity boundary conditions of drug flux and concentration are applied at the interface boundaries between the various regions. The continuity boundary conditions are applied for both free and bound DOX as

29 $$ \left. {\left( { - D_{aj} \nabla C_{j} + v_{i} C_{j} } \right)} \right|_{{r = r_{a}^{ - } }} = \left. {\left( { - D_{tj} \nabla C_{j} + v_{i} C_{j} } \right)} \right|_{{r = r_{a}^{ + } }} $$

30 $$ \left. {\left( { - D_{tj} \nabla C_{j} + v_{i} C_{j} } \right)} \right|_{{r = r_{t}^{ - } }} = \left. {\left( { - D_{nj} \nabla C_{j} + v_{i} C_{j} } \right)} \right|_{{r = r_{t}^{ + } }} $$

31 $$ \left. {C_{j} } \right|_{{r = r_{a}^{ - } }} = \left. {C_{j} } \right|_{{r = r_{a}^{ + } }} $$

32 $$ \left. {C_{j} } \right|_{{r = r_{t}^{ - } }} = \left. {C_{j} } \right|_{{r = r_{t}^{ + } }} $$

where $\nabla$ is the gradient operator in the spherical coordinate system and the index *j* is used to indicate either the free or bound DOX.

No flux (Neumann) boundary condition is applied at the outer boundary of the normal region as

33 $$ \left. {\left( { - D_{nj} \nabla C_{j} + v_{i} C_{j} } \right)} \right|_{{r = r_{n} }} = 0 $$

The release process of DOX drug from the implant surface is modelled using the following flux boundary condition:

34 $$ \left. { - D_{doxi} \nabla C_{exi} } \right|_{{r = r_{0} }} = F_{rs} (t) $$

where *F*_*rs*_(*t*) is given by Eq. (21).
